# *Bacillus cereus* Decreases NHE and CLO Exotoxin Synthesis to Maintain Appropriate Proteome Dynamics During Growth at Low Temperature

**DOI:** 10.3390/toxins12100645

**Published:** 2020-10-06

**Authors:** Catherine Duport, Ludivine Rousset, Béatrice Alpha-Bazin, Jean Armengaud

**Affiliations:** 1 Avignon University, Biology Department, INRAE, UMR SQPOV, F-84914 Avignon, France; ludivine.rousset@inra.fr; 2 Département Médicaments et Technologies pour la Santé (DMTS), Université Paris Saclay, CEA, INRAE, SPI, 30200 Bagnols-sur-Cèze, France; beatrice.alpha-bazin@cea.fr (B.A.-B.); jean.armengaud@cea.fr (J.A.)

**Keywords:** *Bacillus cereus*, shotgun proteomics, exotoxins, low-temperature

## Abstract

Cellular proteomes and exoproteomes are dynamic, allowing pathogens to respond to environmental conditions to sustain growth and virulence. *Bacillus cereus* is an important food-borne pathogen causing intoxication via emetic toxin and/or multiple protein exotoxins. Here, we compared the dynamics of the cellular proteome and exoproteome of emetic *B. cereus* cells grown at low (16 °C) and high (30 °C) temperature. Tandem mass spectrometry (MS/MS)-based shotgun proteomics analysis identified 2063 cellular proteins and 900 extracellular proteins. Hierarchical clustering following principal component analysis indicated that in *B. cereus* the abundance of a subset of these proteins—including cold-stress responders, and exotoxins non-hemolytic enterotoxin (NHE) and hemolysin I (cereolysin O (CLO))—decreased at low temperature, and that this subset governs the dynamics of the cellular proteome. NHE, and to a lesser extent CLO, also contributed significantly to exoproteome dynamics; with decreased abundances in the low-temperature exoproteome, especially in late growth stages. Our data therefore indicate that *B. cereus* may reduce its production of secreted protein toxins to maintain appropriate proteome dynamics, perhaps using catabolite repression to conserve energy for growth in cold-stress conditions, at the expense of virulence.

## 1. Introduction

Bacterial pathogens often have to deal with abiotic stress, which threatens their proliferation and survival [1,2]. Most strains have developed a complex molecular strategy, in which proteins play a crucial role, to rapidly respond to stress [3]. Proteins contribute to physiological and phenotypic changes that are essential for this adaptation and are key contributors to maintaining cellular homeostasis [4]. Generally, proteins do not contribute individually to the dynamic regulation of cellular processes; rather, they operate within complex proteomic networks. Mass spectrometry (MS)-based proteomics, in particular shotgun proteomics approaches, is a powerful means to decipher proteomic networks and gain an overall view of the molecular changes governing adaptation of pathogens to abiotic stress [5].

Pathogens must deal with various types of abiotic stress, including cold stress upon exposure to low temperatures [6]. This is the case for the pathogens in the *Bacillus cereus sensu lato* (sl) group, particularly *B. cereus*, *B. anthracis*, *B. thuringiensis*, and *B. cytotoxicus*, due to their living conditions [7,8,9].

*B. cereus* causes food poisoning and local and systemic infections in humans [10] and has been linked to two well-studied syndromes associated with food poisoning: the diarrheal and the emetic syndromes. Several exoproteins—such as the pore-forming toxins hemolysin BL (HBL), non-hemolytic enterotoxin (NHE), cytotoxin K (CytK), and hemolysin I (CLO), as well as phospholipases, proteases, and the EntD protein—could contribute to the diarrheal syndrome [10,11,12,13]. In contrast, the emetic syndrome is caused by a single depsipeptide toxin (cereulide), the biosynthetic machinery for which is encoded by genes localized on the pCER270 megaplasmid [14]. Similarly, *B. cytotoxicus* has been linked to severe foodborne diarrheal outbreaks and possesses the *cytK*-1 variant of the gene encoding CytK [15,16]. *B. anthracis* is the agent responsible for anthrax, a highly lethal infectious disease in humans [17]. Its pathogenicity is generally attributed to the production of a poly-γ-d-glutamic acid capsule and anthrax exotoxins, the genes for which are present on the plasmids pXO1 and pXO2 [18]. However, other secreted proteins—including proteases, phospholipases, and toxins from the cholesterol-dependent cytolysin (CDC) family, such as anthrolysin O (ALO, [19,20,21,22]), which is very similar to *B. cereus* CLO—could also be pathogenic factors. *B. thuringiensis* differs from the other pathogenic *B. cereus sl* species through its ability to produce crystalline parasporal inclusions during sporulation. These inclusions contain two types of ∂-endotoxins: Cry and Cyt proteins, which have insecticidal properties [23,24]. Some *B. thuringiensis* strains secrete additional proteins which further increase their entomopathogenicity [25]. Like *B. cereus* and *B. anthracis*, *B. thuringiensis* chromosomes carry genes encoding enterotoxins and other known cytotoxic virulence factors. However, its human pathogenicity remains controversial [26].

A phylogenetic analysis of 425 strains divided the *B. cereus sl* group into seven major subgroups, distinct from the eight-species delineation [8,9]. Each subgroup was associated with a well-defined growth temperature range and thus constituted a thermal group (thermotype). Unlike *B. cytotoxicus* (group VII, 20–50 °C) and *B. anthracis* (group III, 15–45 °C), *B. cereus* and *B. thuringensis* species are distributed across several thermal groups. Interestingly, the emetic *B. cereus* strains are grouped in the same mesophilic group as *B. anthracis* strains, reflecting their extensive similarities. The ability of *B. cereus sl* strains to adapt to low-temperature conditions correlates with their thermotype and at the molecular level depends on the two-component CasK/R system, which perceives and transduces the signal to regulate the genes encoding proteins involved in fatty acid metabolism [27]. As in other species, *B. cereus* cold adaptation includes proteins that function as RNA chaperones—such as RNA helicase [28] and cold-shock proteins (CSP) [29]—as well as proteins involved in replication, DNA repair and maintenance, transcription, and translation [6].

The impact of cold stress on toxin production has been little documented until now, and we lack information on the overall molecular dynamics. Generally, high growth temperatures favor increased production of enterotoxins and cereulide in mesophilic strains. However, no clear correlation has been established between growth temperature and toxin production [30,31]. The temperature-dependence of toxin production is even less evident in psychotropic strains, which produce similar levels of enterotoxins at low and high temperatures [32], while producing low levels of cereulide [33].

In this study, we compared the dynamics of the cellular proteome and exoproteome for the emetic *B. cereus* AH187 (F4810/72) strain grown at low (16 °C) and high (30 °C) temperatures. Our results reveal that protein toxins contribute significantly to temperature-induced proteome changes in *B. cereus* and provide new molecular evidence enhancing our understanding of the temperature-dependent regulation of their production.

## 2. Results

### 2.1. Physiological Changes Induced by Low Temperature

**Figure 1:**
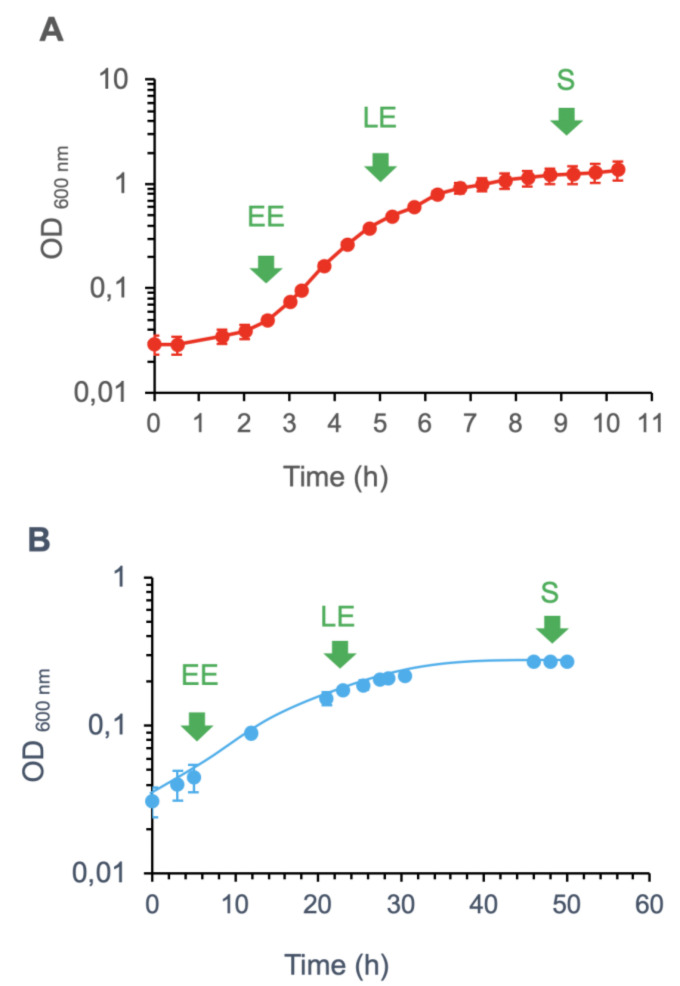
Growth curves at 30 °C (**A**) and 16 °C (**B**) for *B. cereus* in MOD medium supplemented with 30 mM glucose. Values correspond to mean ± SD measured for three biological replicates. Samples for proteomics analyses were harvested at early exponential (EE), late exponential (LE), and stationary (S) growth phases, as indicated.

*B. cereus* AH187 cells were cultivated in aerobic conditions on glucose-supplemented MOD medium at 16 °C or 30 °C. Figure 1 shows that cultures grew more slowly at 16 °C (µ_max_ = 0.09 ± 0.01 h^−1^) than at 30 °C (µ_max_ = 0.9 ± 0.1 h^−1^), and produced a significantly lower final biomass (OD_600_ = 0.27 ± 0.01 compared to 1.4 ± 0.3, respectively). To link growth to a physiological response, we determined the glucose uptake rate at µ_max_ (i.e., during the EE growth-phase, at OD_600_ = 0.05 for the two conditions). Cells grown at 30 °C had a maximal glycolytic activity of approximately 9 mmol/h, which increased to approximately 228 mmol/h at 16 °C. These values indicate increased glycolytic flow at 16 °C, which probably compensates for decreased activity of glycolytic enzymes [34].

**Figure 2:**
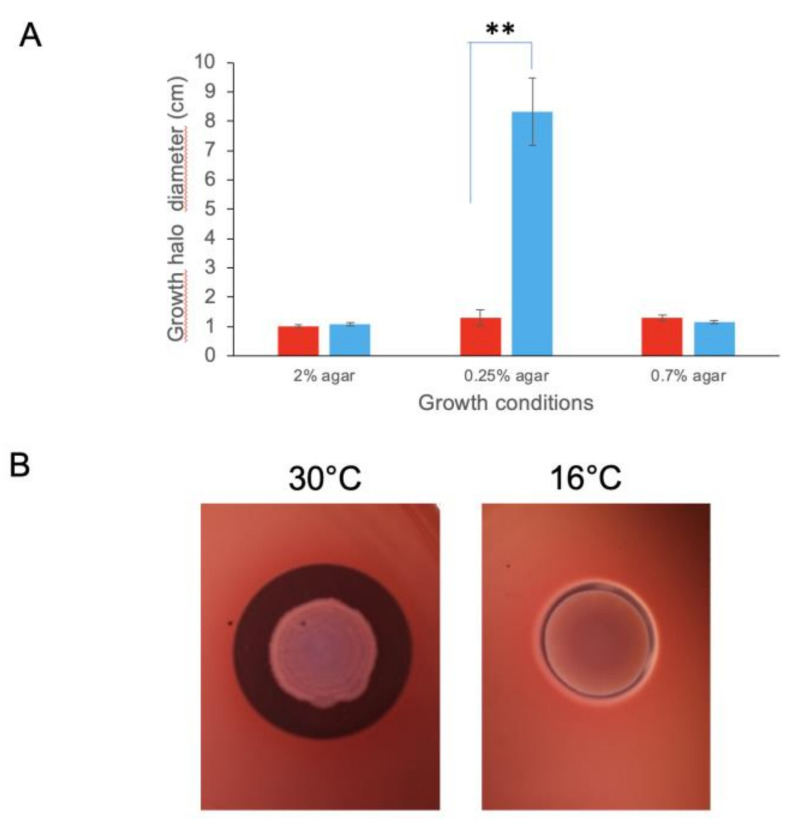
*B. cereus* phenotypes at high (30 °C) and low (16 °C) temperatures. (**A**) Motility of *B. cereus*. Swimming and swarming motilities were tested using TrB medium containing 0.25 and 0.7% agar at 30 °C (red) and 16 °C (blue). Control plates contained TrB medium with 2% agar. ** *p*-value < 0.01 according to Student’s *t*-test. (**B**) *B. cereus* hemolytic activity. Assays were performed on blood agar plates incubated at 30 °C and 16 °C.

We then determined the effect of the low-temperature condition on the capacity of *B. cereus* to form heat-resistant endospores and produce biofilm. No difference between the two temperature conditions was observed (data not shown). We also compared motility of *B. cereus* after 8- and 12-days of incubation on TrB medium at 30 °C and 16 °C, respectively. In these conditions, growth halos had similar diameters on TrB medium solidified with 2% agar (control) and 0.7% agar. In contrast, when cultures were grown on TrB medium solidified with 0.25% agar, the growth halo diameter was significantly smaller at 16 °C than at 30 °C (Figure 2A). Thus, *B. cereus* cells had lower swimming motility at 16 °C than at 30 °C.

We also analyzed the hemolytic activity of *B. cereus* by incubating cultures on blood agar plates at 16 °C and 30 °C, and measuring the diameters of clear zones around colonies. Results from these assays showed that *B. cereus* had decreased hemolytic activity at 16 °C compared to 30 °C (Figure 2B).

In summary, exposure to low temperature caused *B. cereus* AH187 to decrease its growth rate, swimming motility, and hemolytic activity compared to equivalent cultures grown at high temperature.

### 2.2. Cellular Proteome Dynamics

Cell samples were collected at the three growth phases indicated in Figure 1—EE, LE, and S—and used for proteomics analysis. Biological triplicates were performed, generating 18 samples in total (9 samples each for the 2 growth temperatures). Overall, our shotgun analysis identified 2,302 cellular proteins, each of which was associated with at least two distinct peptides. Proteomics analysis thus produced a dataset (Table S1) composed of 2063 elements (proteins quantified by their relative normalized spectral abundance factor (NSAF)) with 18 variables (proteomes for one time-point in one temperature condition).

**Figure 3:**
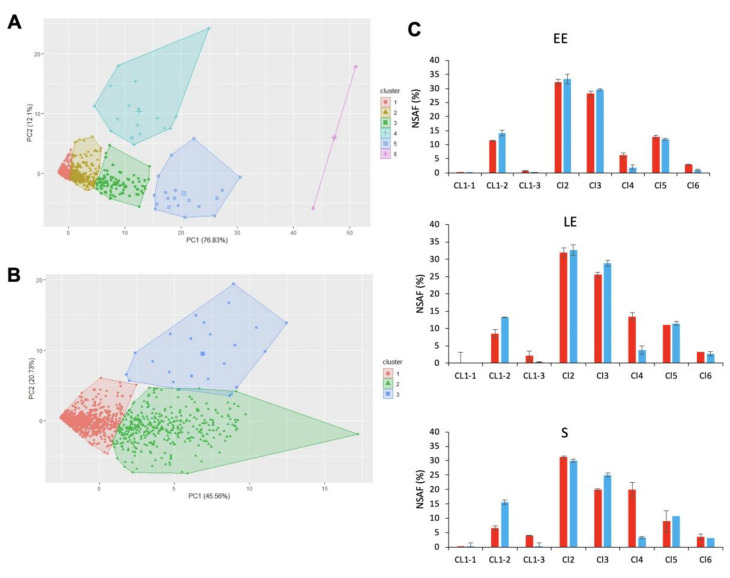
Hierarchical clustering on principal components (HCPC) for *B. cereus* cellular proteins. (**A**) Principal component (PC) analysis of 30 °C- and 16 °C-proteomes for *B. cereus* cells harvested at early exponential (EE), late exponential (LE), and stationary (S) growth phases. Proteins are represented by colored marks and are plotted as a function of their PC1 and PC2 values. The colors applied to proteins were based on HCPC clustering, which produced a total of six clusters. (**B**) HCPC sub-clustering of proteins in cluster 1. The three subclusters are distinguished by color, and the proteins were plotted as a function of their PC1 and PC2 values. (**C**) Protein abundance levels (% NASF) associated with each cluster (CL) for EE, LE, and S growth phases at 30 °C (red) and 16 °C (blue). Values correspond to mean ± SD for three biological replicates.

To focus on proteins that contributed the most to dataset variability, we performed principal component analysis (PCA) followed by hierarchical clustering on principal components (HCPC). PCA results showed that the first two principal components, PC1 and PC2, explained 76.83% and 12.1% of total data variability, respectively. PC1 did not distinguish the proteomes that are described by proteins with differences in abundance levels. PC2 opposed the 30 °C-proteomes (positive values on PC2) to the 16 °C-proteomes (negative values on PC2). HCPC grouped proteins into six clusters (CL, Table S2), which were projected into the PC1–PC2 space to produce Figure 3A. Among these six clusters, CL4 was identified as the main contributor to the differences between 30 °C- and 16 °C-cellular proteomes. This cluster comprised 18 medium-abundance proteins (mean NSAF value = 0.45 ± 0.46%), which were detected at higher levels in cells grown at 30 °C than at 16 °C (Figure 3A). Taken together, based on their cumulated NSAF, these 18 proteins accounted for up to 20% of the total cellular proteome at 30 °C (Figure 3C). Interestingly, CL6 contained two highly-abundant cold-shock proteins (very high coordinates on PC1), one of which (CspB2, B7HZX9) also had a high positive coordinate on PC2, whereas the other (CspA1, B7HZQ7) had a negative coordinate on PC2. This difference in profiles indicates that CspB2 was more abundant at 30 °C than at 16 °C, whereas CspA1 was more abundant at 16 °C than at 30 °C.

In contrast to the small number of proteins contained in other clusters, a very large proportion of all the proteins detected (80%, i.e., 1604 proteins) was included in CL1. Overall, these proteins were low-abundance proteins (mean NSAF value = 0.01 ± 0.08%), explaining why their mean coordinate was negative on PC1 (Figure 3A). To identify the proteins that had the highest variability within CL1, we performed a second PCA/HCPC on the CL1 dataset for the 18 proteomes. This analysis yielded three CL1 subclusters, with the greatest variability observed for CL1–3 (Figure 3B). This subcluster consisted of 21 proteins, detected at higher levels in samples from 30 °C-cultures than from 16 °C-cultures, whatever the growth-phase (Figure 3B). Collectively, CL1–3 proteins were more abundant in S-phase samples than in LE- and EE-phase samples, whatever the temperature (Figure 3C).

**Figure 4:**
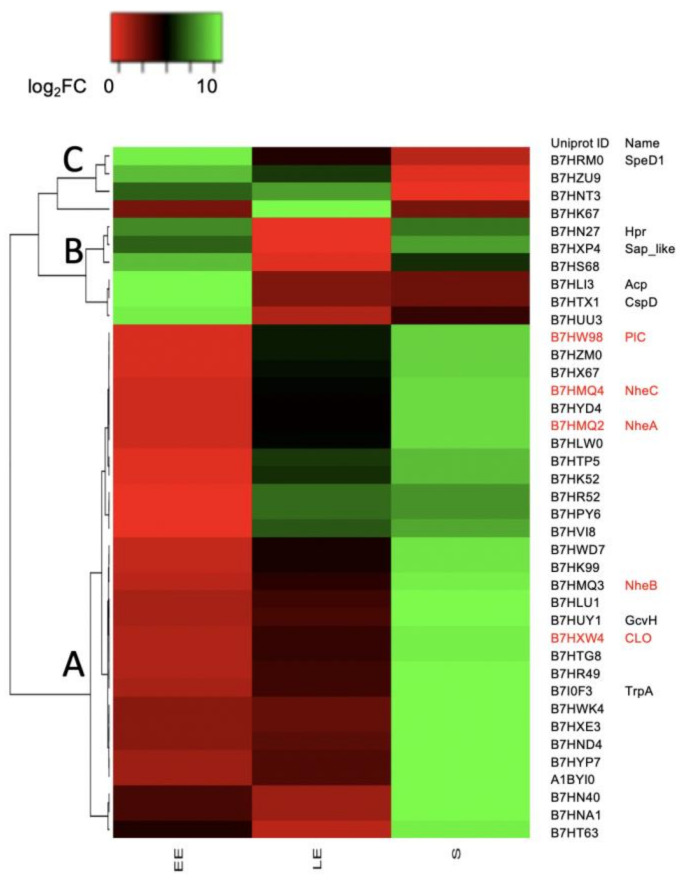
Heatmap showing log_2_ fold change (FC) values for cellular proteins included in clusters CL1–4 and CL3. FC is the ratio of protein abundance measured for samples from 30 °C cultures over abundance levels measured for 16 °C cultures. Green indicates a high FC, whereas red corresponds to a low FC in the early exponential (EE), late exponential (LE), and stationary (S) growth phases. Proteins on the right were hierarchically clustered.

Comparison of all clusters revealed that proteins co-clustered in CL4 and CL1–3 responded most to temperature changes during growth. We therefore focused our attention on these 39 proteins. We first assigned the 39 proteins to clusters of orthologous groups (COG) (Table 1) and compared their relative abundances (log_2_ fold-change (FC) for abundance levels determined at 16 °C relative to at 30 °C) for each growth-phase, using a heatmap (Figure 4). Hierarchical clustering identified three distinct groups. (i) Group A comprised 29 proteins with lower log_2_FC in EE- compared to S-phase samples, i.e., their abundance levels increased more strongly during growth at 30 °C than at 16 °C. These proteins are mainly cell-surface-associated and extracellular proteins (Table 1).

Among them, two proteins are components of the flagellar apparatus, two (B7HVI8 and B7HXE3) share extensive sequence identity with the *B. cereus* ATCC 14579 putative virulence factors EntA and EntC [12,35], four have S-layer domains, and five are exotoxins (NHE enterotoxin components—the abundant NheA and NheB components, and the low-abundance NheC component [36]—the low-abundance zinc-dependent phospholipase C (PlC), which is also recognized as an alpha toxin [37], and the low-abundance hemolysin CLO). These exotoxins co-clustered with B7HPY6, a putative small XRE transcriptional regulator. (ii) Proteins in group B had a higher log_2_FC in the EE growth-phase than proteins in group A. Their log_2_FC decreased in the LE-phase, indicating that their abundance increased more rapidly at 16 °C than at 30 °C during active growth. The log_2_FC stayed low in the S-phase for CspD and AcpP and increased for Hpr and B7HXP4, and to a lesser extent for B7HUU3 and B7HS68 (Figure 4). Interestingly, AcpP is a predicted acyl carrier protein linked to fatty acid synthesis regulation in response to cold stress [38], and CspD is a cold-shock protein [38]. The phosphocarrier protein Hpr is an essential component of the sugar-transporting phosphotransferase system (PTS) and plays an important role in regulating carbohydrate and energy metabolism [39]. B7HXP4 shares extensive sequence similarity with the S-layer Sap protein from *B. anthracis* [40]; B7HUU3 is a NifU-containing protein that may contribute to Fe-S cluster assembly in proteins, mainly at low temperatures; and B7HS68 is a predicted component of pathways controlling lipid transport and metabolism. (iii) Group C was distinguished from the other groups by a high log_2_FC in S-phase samples. With the exclusion of SpeD1, this group consisted only of uncharacterized proteins.

In summary, our data indicate that the temperature-dependent abundance changes for proteins in group A, in particular protein exotoxins (Nhe ABC, CLO, PlC) correlated positively with changes in abundance recorded for the putative regulator B7HPY6, and negatively with changes in abundance measured for the regulators CspD and Hpr during active growth.

### 2.3. Exoproteome Dynamics

**Figure 5:**
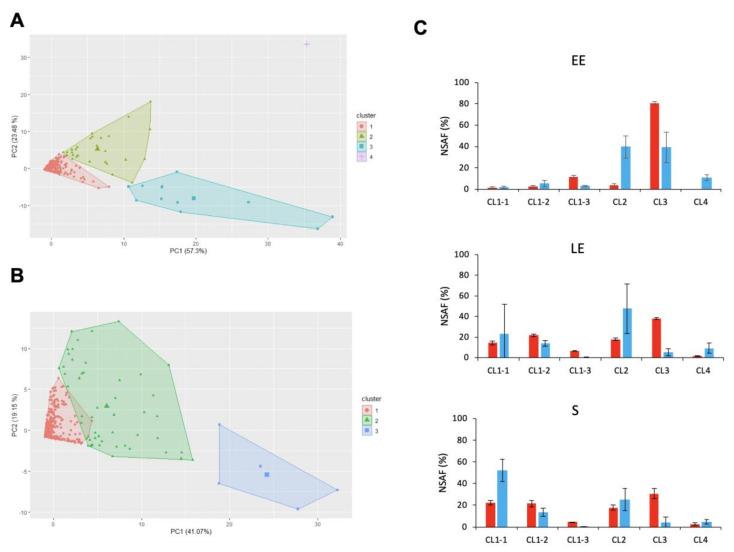
Hierarchical clustering of *B. cereus* extracellular proteins. (**A**) Principal component (PC) analysis of 30 °C and 16 °C-exoproteomes for *B. cereus* cells harvested at early exponential (EE), late exponential (LE), and stationary (S) growth phases. Individual exoproteins are represented by colored marks and are plotted as a function of their PC1 and PC2 values. Proteins were colored based on HCPC clustering, which produced four clusters, as shown. (**B)** HCPC sub-clustering of cluster 1. The three subclusters were attributed distinguishing colors, and the proteins are plotted as a function of their PC1 and PC2 values. (**C**) Exoprotein abundance levels (% NASF) associated with each cluster (CL) at EE, LE, and S growth phases at 30 °C (red) and 16 °C (blue). Values correspond to mean ± SD for three biological replicates.

A total of 900 proteins were identified in the *B. cereus* exoproteome, based on the confident detection of at least two distinct peptides for each protein (Table S3). HCPC analysis of this dataset x 18 proteomes yielded four clusters (Table S4), the projection of which in the PC1–PC2 plane is shown in Figure 5A. PC1 explained the variability of 30 °C-exoproteomes (57.3%), and PC2 the variability of 16 °C-exoproteomes (23.5%). CL3 was identified as the main contributor to the variability of both 30 °C- and 16 °C-proteomes. The 12 proteins in CL3 were more abundant at 30 °C than at 16 °C (Figure 5A), and their abundances combined corresponded to up to 80% of the total EE-exoproteome at 30 °C (Figure 5C).

Interestingly, we noted that CL4, which had a high positive coordinate in the PC1–PC2 space in Figure 5A, only contained a single protease, B7HVA4. The abundance of B7HVA4 was significantly higher at 16 °C than at 30 °C for the EE and LE growth phases (log_2_FC = 5.6 and 2.5, respectively, *p* < 0.05). Once again, a single group, CL1, gathered together the vast majority of the exoproteins identified. To analyze these 850 low-abundance proteins in more detail, we applied a second HCPC analysis that yielded three subclusters (Figure 5B). CL1–3 comprised five proteins, for which abundance levels were higher at 30 °C than at 16 °C. As for CL3, the abundance levels for proteins in CL1–3 decreased as growth progressed, both at 30 °C and 16 °C (Figure 5C). The decrease in abundance levels measured for proteins in CL3 and CL1–3 during the LE and S growth phases compared to the EE-phase was offset by the increase in abundance measured for proteins in CL2 and CL1–1, CL1–2, respectively, particularly at 30 °C (Figure 5C).

The 17 proteins that co-clustered in CL1–3 and CL3 are listed in Table 2. These proteins correspond to cell-surface-associated proteins, proteases, and toxins. Of these 17 proteins, 9 were identified as cellular proteome contributors, as detailed above. These proteins were the flagellin B7HLW0; the proteases A1BYI0, B7HND4, and B7HTG8; the putative cell wall peptidase B7HNA1; EntC (B7HXE3); and the three exotoxins, NheA, NheB, and CLO. In contrast to our results for the cellular proteome, PlC and NheC did not co-cluster with CLO in the exoproteome (Table S4), as PlC and NheC were undetected in exoproteome samples for EE-phase cells at both 30 °C and 16 °C (Table S3). However, both proteins were significantly more abundant in exoproteomes from the LE- and S-phases at 30 °C compared to 16 °C (log_2_FC = 2.9 and 5.5 at LE and S, respectively, for NHE; 4.5 and 5.5 for PlC). While HCPC analysis identified the Sap-like protein B7HXP4 as a major contributor to cellular proteome adaptation, it identified the putative second S-layer component EA1 (B7HXP5) as a major contributor to alterations to the exoproteome between 30 °C and 16 °C (Table 2). Unlike EA1, B7HXP4 was undetected in 16 °C-exoproteomes (Table S3) and consequently did not contribute to 16 °C-exoproteome dynamics.

## 3. Discussion

The aim of this study was to determine the changes occurring in the cellular- and exo-proteomes of an emetic *B. cereus* strain grown at 30 °C or 16 °C. In pathogens, cellular proteome and exoproteome dynamics depend on numerous regulatory processes (transcription, translation, secretion, proteolysis) as well as on the biochemical properties of the proteins with altered expression levels. By elucidating the dynamics of the cellular and exoproteomes, it is thus possible to identify proteins that play a role in proteome homeostasis, and consequently in the response mounted by pathogens to environmental stressors. The results presented here indicate that exotoxins play a role in controlling homeostasis of the *B. cereus* cellular proteome and exoproteome in response to cold stress.

Protein exotoxins, particularly the NHE components, CLO and PlC, accumulated in the cellular proteome during growth at 30 °C, especially during the transition from log-phase to stationary-phase growth. This accumulation was mainly the result of upregulated transcription and involves several sensors/regulators that sense growth perturbations [10,14,30]. In response to growth at a low temperature, *B. cereus* accumulated lower levels of toxins. This response was associated with changes to several cellular processes and/or regulatory pathways, as revealed by hierarchical clustering analysis. (i) The decrease in toxin abundance correlates with decreased expression of the proteins Hpr and AcpP, which are well-known regulators of carbohydrate/energy and lipid homeostasis, respectively [41]. Interestingly, phosphorylated Hpr (Hpr-Ser~P) is an effector of the CcpA transcriptional regulator [42]. The Hpr-Ser~P-CcpA complex binds to DNA-target sites known as catabolite response elements (CREs). Two CRE sites through which CcpA/Hpr-Ser~P binding mediates gene repression were identified in the *nhe* enterotoxin operon in *B. cereus* ATCC 14579 [43]. These sites are also present in the *nhe* operon in *B. cereus* AH187, and we identified additional putative CRE sites in the promoter regions of the genes encoding the toxins CLO and PlC (Supplementary Material- Figure S1). The accumulation rate for Hpr during active growth (i.e., between the EE and LE growth phases) was higher at 16 °C than at 30 °C, a result that is probably linked to the increase in glycolytic flow measured. Therefore, Hpr/CcpA complex-mediated repression of toxin gene expression may be higher at low temperature compared to high temperature. (ii) Intracellular toxin abundance correlated with levels of the cold-shock protein CspD in a growth phase- and temperature-dependent manner. In *E. coli*, CspD functions as an S-phase-induced stress response protein, inhibiting DNA replication without binding to a specific recognized sequence [44]. Like its paralog in *E. coli*, CspD in *B. cereus* reached its maximum concentration during the S-phase of growth in both temperature conditions. In addition, its growth-phase-dependent abundance change was higher in the low-temperature condition than in the high-temperature condition, suggesting a greater contribution of CspD to growth-phase-dependent regulation at low temperature. (iii) The decrease in exotoxin abundance at 16 °C was associated with reduced levels of flagellar components, confirming the existence of a link between toxinogenesis and flagella synthesis [45]. (iv) Exotoxin abundance was also observed to be linked to the level of Sap-like components in the S-layer, suggesting a shared regulatory pathway in response to low temperatures. In summary, *B. cereus* could limit accumulation of exotoxins and flagellar components, as well as one S-layer component, as a result of CcpA-mediated catabolite repression. We also identified a putative transcriptional regulator (B7HPY6), levels of which positively correlated with the dynamic changes in exotoxin levels. B7HPY6, together with CspD, could thus be an interesting new candidate member of the very complex regulatory network modulating toxin synthesis [46].

The synthesis of exotoxins and surface proteins, especially proteins anchored to the bacterial surface, including flagella, is energetically costly. By decreasing synthesis of these proteins and of proteins controlling growth and biomass production, *B cereus* conserves its energy for processes essential to survival in a cold stress context. The physiological consequences of these changes are slowed growth and loss of motility, as observed here, and possibly attenuated pathogenicity.

Pathogenicity depends on the accumulation of toxins and other virulence factors in the extracellular medium, and thus in the exoproteome. To accumulate in the exoproteome, proteins must be synthesized within the cells, secreted by appropriate pathways, and/or be resistant to proteolysis. Our results indicated that the toxin content in the *B. cereus* exoproteome decreased when cells were grown at low temperature compared to high temperature; in addition, abundance levels were lower for cells in the S-phase compared to the log-phase of growth, whatever the culture temperature. Cellular proteome analysis revealed that the abundance of AcpP and other cell-surface components was reduced at low- compared to high-temperature, suggesting alterations to membrane fluidity, active transport, and thus secretory capacity [47]. Analysis of exoproteome data indicated that proteolysis, possibly mediated by the highly-abundant extracellular protease B7HVA4, could play a role in the temperature- and growth-phase-dependent decrease in toxin abundance levels. Finally, the toxin-depletion observed in the low-temperature exoproteome could be the result of decreased toxin synthesis due to catabolite repression combined with an altered secretory capacity and a higher rate of proteolysis.

The protein toxins detected at lower abundance in low-temperature samples included CLO, which is hemolytic [48]. As hemolytic activity contributes to *B. cereus* pathogenicity, we measured it in our different temperature conditions. The fall-off in hemolytic activity observed for *B. cereus* AH187 at 16 °C could be explained by both the reduced extracellular concentration of CLO in these conditions. However, other extracellular proteins may also contribute to the decrease in hemolytic activity observed [49].

In conclusion, the results of our proteomics study show that exotoxins were produced at lower levels by *B. cereus* AH187 in low-temperature cultures compared to high-temperature cultures. By reducing production of secreted toxins and other non-essential secreted proteins, *B. cereus* probably conserves energy to devote to overcoming cold stress. In addition to highlighting the role of toxins as cold stress responders, the results presented here demonstrate the power of shotgun proteomics methods to identify novel candidate regulators of toxinogenesis. Overall, this study represents a comprehensive analysis at the cellular proteome and exoproteome levels of *B. cereus* grown at optimal and low temperature. However, further studies with a representative number of *B. cereus sl* group strains are required to grasp the complexity of the cold stress response.

## 4. Material and Methods

### 4.1. Culture Conditions and Proteomics Sample Preparation

*B. cereus* AH187 (F4872/10) was grown in MOD medium supplemented with 30 mM glucose and buffered at pH 7. Cultures were grown at 30 °C or 16 °C under continuous shaking (200 rpm). MOD medium (200 mL in 2 L Erlenmeyer flasks) was inoculated at an OD_600_ of 0.02 from overnight precultures. Samples (50 mL) were collected at early exponential (EE), late exponential (LE), and stationary (S) growth phases, as indicated in Figure 1. Protein extracts were prepared from cell pellets and culture supernatants as previously described [50,51]. Protein samples were loaded onto NuPAGE Bis–Tris 4–12% gels (Invitrogen) for a short 5 min migration at 200 V [52], and then digested in-gel with mass spectrometry grade Trypsin Gold (Promega) in the presence of 0.01% ProteaseMAX surfactant (Promega). Triplicate samples were prepared for each culture condition.

### 4.2. NanoLC/MS-MS Analysis

A Q Exactive HF tandem mass spectrometer coupled to an UltiMate 3000 LC system (Dionex-LC Packings) was used to analyze peptide mixtures resulting from trypsin proteolysis. Analysis started with desalting of peptide mixtures on a reverse-phase precolumn (Acclaim PepMap 100 C18 column; 5 μm, 100 Å, 300 μm id, × 5 mm), followed by peptide separation at a flow rate of 0.2 μL/min on a reverse-phase Acclaim PepMap 100 C18 column (3 μm, 100 Å, 75 μm id, ×500 mm). Solvent A was 0.1% formic acid in water, and solvent B was 0.1% formic acid and 80% CH_3_CN. Peptides obtained from cellular proteins were eluted using a 75 min gradient of solvent B (4–25%); the solvent B concentration was then ramped up to 40% for 15 min. Peptides obtained from extracellular proteins were eluted using a 2.5–25% gradient of solvent B over 50 min, followed by 40% for 10 min. The instrument was operated in data-dependent mode at a resolution of 60,000 to determine the masses of peptides, and a Top20 method with a 10 s dynamic exclusion was applied to select peptides for fragmentation [53].

MS/MS spectra were searched against the *B. cereus* AH187 NCBI_20180517 database using MASCOT Daemon software (Version 2.5.1; Matrix Science). The search algorithm parameters were as follows: allow a maximum of two missed cleavages, cysteine carbamidomethylation as fixed modification, methionine oxidation as variable modification, and a precursor mass tolerance of 5 ppm for peptides and 0.02 Da for MS/MS fragments. Peptide-to-spectrum matches were assigned when the *p*-value was below 0.05 in identity threshold mode. Proteins were validated when identified by at least two distinct peptide sequences.

The mass spectrometry proteomics data have been submitted to the ProteomeXchange Consortium via the PRIDE [54] partner repository, under dataset identifiers PXD020456 (DOI: 10.6019/PXD020456) and PXD020463 (DOI: 10.6019/PXD020463).

### 4.3. Clustering and Statistical Analyses

Hierarchical clustering on principal components (HCPC) was performed using the principal component analysis (PCA) function in the FactomineR package for R [55], followed by the HCPC function in the same package [56]. Data were autoscaled, and all other options were used at their default settings. The HCPC tree was cut at the recommended level to maximize inertia gain.

To determine significant differences in protein amounts at 30 °C compared to 16 °C, we used the limma package for R [57].

### 4.4. Analytical Procedures and Phenotypic Characterization

To assay glycolytic flow, glucose concentrations were determined from filtered culture supernatants using the Enzytec Fluid kits purchased from R-Biofarm, as recommended by the manufacturer.

Swimming and swarming motility assays were performed as described previously in tryptone-NaCl (TrB) medium (1% tryptone, 0.5% NaCl) [12,58].

Hemolytic activity was tested on sheep blood agar plates (tryptic soy agar supplemented with 5% sheep blood, Biomerieux) incubated for 24 h at 30 °C or for 48 h at 16 °C. The diameter of clear zones around colonies was measured as an indication of hemolytic activity. Data from three independent biological replicates were analyzed, and statistical significance was assessed by applying a Student’s *t*-test.

## Figures and Tables

**Table 1:** Components of cellular proteome classified in clusters CL1–3 (blue) and CL4 (red) and their differential accumulation between 30 °C and 16 °C.

Category	Uniprot ID	Protein Name	Function	Subcellular Localization	Log_2_FC *		
					EE	LE	S
Amino acid metabolism	B7HRM0	SpeD1	S-adenosylmethionine decarboxylase proenzyme	Cytoplasm	5.5	3.8	2.7
	B7HUY1	GcvH	Glycine cleavage system H protein	Cytoplasm	1.3	1.7	2.7
	B7I0F3	TrpA	Tryptophan synthase alpha chain	Cytoplasm	1.3	1.9	3.4
Carbohydrate transport and metabolism	B7HN27	Hpr	Phosphocarrier protein	Cytoplasm	2.5	1.9	2.5
Cell motility	B7HLW0		Flagellin	Extracellular	2.3	3.9	5.5
	B7HLU1	HAP2	Flagellar hook-associated protein 2	Extracellular	1.4	3.1	7.9
Cell wall, membrane, envelope biogenesis	B7HXP4	Sap-like	Crystal protein	Cell wall	3.3	2.5	3.4
	B7HZM0		Putative S-layer protein	Cell wall	1.1	2.9	4.3
	B7HVI8	EntA	Enterotoxin	Cell wall	1.0	5.9	7.0
	B7HWK4		S-layer domain protein	Cell wall	2.7	3.0	5.8
	B7HXE3	EntC	Enterotoxin cell wall binding protein	Cell wall	1.7	1.8	2.6
	B7HYP7		S-layer domain protein	Cell wall	2.9	3.4	5.3
	B7HK52		Putative internalin	Cell wall	1.6	4.7	6.2
	B7HNA1		Putative cell wall peptidase	Cell wall	2.2	1.9	3.5
Function unknown	B7HR52		Uncharacterized protein	Cytoplasm	1.0	5.1	5.6
	B7HZU9		Uncharacterized protein	Cytoplasm	2.8	2.2	0.7
	B7HNT3		Uncharacterized protein	Cytoplasm	5.9	6.5	2.0
	B7HK67		Conserved domain protein	Cytoplasm	4.5	4.8	4.5
	B7HTP5		Uncharacterized protein	Cytoplasm	1.9	5.3	6.7
	B7HR49		MbtH-like protein	Cytoplasm	3.2	4.3	6.9
	B7HT63		Uncharacterized protein	Membrane	3.3	0.1	8.3
General function only	B7HK99		Flavodoxin	Cytoplasm	1.5	2.6	4.1
	B7HWD7		Ferrous iron transport protein A	Cytoplasm	0.6	1.4	2.5
Inorganic ion transport and metabolism	B7HS68		Lipoyl-binding domain-containing protein	Cytoplasm	3.0	0.8	2.3
Lipid transport and metabolism	B7HLI3	Acp	Acyl carrier protein	Cytoplasm	4.6	1.9	2.1
	B7HX67		Lipoteichoic acid synthase	Cytoplasm	–1.0	3.3	6.8
	B7HYD4		Glycerophosphoryl diester phosphodiesterase	Cytoplasm	NS	1.8	3.3
	B7HUU3		NifU domain protein	Cytoplasm	2.0	1.2	1.4
Posttranslational modification, protein turnover, chaperones	B7HND4		Putative protease	Extracellular	5.4	5.7	8.7
	B7HTG8		Neutral metalloproteinase	Extracellular	4.8	5.7	8.0
	A1BYI		Neutral metalloproteinase	Extracellular	4.7	5.3	7.8
	B7HN40		Peptidyl-prolyl cis-trans isomerase	Cytoplasm	1.1	0.7	2.2
Toxin (pathogenesis)	B7HW98	PlC	Phospholipase C	Extracellular	2.2	5.5	7.9
	B7HMQ4	NheC	Enterotoxin C	Extracellular	0.9	4.3	7.8
	B7HMQ2	NheA	Enterotoxin A	Extracellular	2.0	5.8	9.7
	B7HMQ3	NheB	Enterotoxin B	Extracellular	2.9	5.4	10.1
	B7HXW4	CLO	Thiol-activated cytolysin	Extracellular	3.8	5.3	9.1
Transcription	B7HTX1	CspD	Cold shock protein	Cytoplasm	3.6	1.1	1.3
	B7HPY6		DNA-binding protein	Cytoplasm	3.4	6.4	6.

* FC: fold-change, i.e., normalized spectral abundance factor (NSAF) ratio between 30 °C and 16 °C. NS: not significant according to statistical criteria (*p* < 0.05, |FC| > 1.5).

**Table 2:** Components of exoproteome classified in cluster CL3 and CL1–3 and their differential accumulation between 30 °C and 16 °C.

Category	Uniprot ID	Protein Name	Function	Log_2_(FC) *		
				EE	LE	S
Cell wall, membrane, envelope biogenesis	B7HNA1		Cell wall peptidase	2.9	4.8	4.7
	B7HX69		Cell wall hydrolase	1.7	2.9	2.5
	B7HX23		Cell wall endopeptidase	NS	5.6	6.1
	B7HXP5	EA1	S-layer crystal protein	NS	1.5	3.4
	B7HXE3	EntC	Enterotoxin	NS	2.6	5.3
Flagella	B7HLW0		Flagellin	2.5	5.0	5.5
Protease	A1BYI0		Neutral metalloproteinase	5.0	4.6	6.4
	B7HKS1		Neutral metalloproteinase	NS	2.9	4.4
	B7HND4		Protease	3.0	4.1	5.2
	B7HTG8		Neutral metalloproteinase	2.5	4.8	6.5
	B7HW94	InhA	Immune inhibitor A metalloprotease	NS	0.6	2.1
Toxin (Pathogenesis)	B7HXW4	CLO	Thiol-activated cytolysin	5.3	3.7	5.5
	B7HMQ2	NheA	Enterotoxin A	2.1	3.8	4.1
	B7HMQ3	NheB	Enterotoxin B	4.2	4.2	5.4
Unknown	B7HXS3		Uncharacterized protein	1.2	5.8	7.2
	B7HWA6		3D domain-containing protein	0.7	3.9	4.7
	A1BYH8		Uncharacterized protein	–1.9	5.0	6.1

* FC: fold-change i.e., NSAF ratio between 30 °C and 16 °C; NS: not significant according to statistical criteria (*p* < 0.05, |FC| > 1.5).

## Supplementary Materials

The following are available online at https://www.mdpi.com/2072-6651/12/10/645/s1, Figure S1: Putative CRE sites, Table S1: NASF values for proteins identified in the cellular proteome of B. cereus AH187 at 30 °C and 16 °C, Table S2: Classification of cellular proteins using hierarchical clustering on principal components, Table S3: NASF values for proteins identified in the exoproteomes of B. cereus AH187 at 16 °C and 30 °C, Table S4: Classification of extracellular proteins using hierarchical clustering on principal components.
