# Supplementary material for: Bacillus cereus Decreases NHE and CLO Exotoxin Synthesis to Maintain Appropriate Proteome Dynamics During Growth at Low Temperature
Source: Toxins (Basel). 2020 Oct 6;12(10):645. doi: 10.3390/toxins12100645 (PMC7601483; doi:10.3390/toxins12100645)
Supplement: Supplementary file 1 [file toxins-12-00645-s001.zip › SUPP conv.pdf]

Supplementary Material: *Bacillus cereus* Decreases Exotoxin Synthesis to Maintain Appropriate Proteome Dynamics During Growth at Low Temperature

Catherine Duport [1,*], Ludivine Rousset [1,†], Béatrice Alpha-Bazin [2] and Jean Armengaud [2]

A.

```
TATAATTTTTGTGTCTGTGAGTAAGTAGAGGTTTTTGTTGTGATATATGGATATTTCAAT
TGAAAAAGTAAAGTGGATTATATTTTAAAATAAAGACATGAAAGCTTGAAAATAAAGGGA
ATGAAAATACTTCTTTACGTTATATAAATAGCTGTGTTTAAATGACCTTACAGTACAAAA
CTTATATGAAAACAAAACGGAATTTCATATTTATGTATGTGAATTAGTTTAAAATGAATA
ATATTTTTATGTAATATATTCACTGTATCAATGTGGGAAATAAGATGAAATAGAAAATTT
ATGCAATGTTATTCATACTAACCACATATGAAAAGTTTTATAACGATAAGTAAGTACTGT
AGCAATAAAATTTGGGAGAGATGGTTCGTGTGTGAATCGCGGTGAGAACGTTCTCTATTT
TAACCATCTCAAACTATGTGCAAGAAAATAGAATAATAAAATGCGATAGTAATAGTTACT
AATACATAGGAGGAGTTAAAGTGAAAAAGACTTTAATTACAGGGTTATTGGTTACAGCAG
TATCTACGAGTTGTTTTATTCCTGTAAGCGCTTACGCTAAGGAGGGGCAAACAGAAGTGA
AAACAGTATATGCACAAAATGTAATTGCTCCAAATACATTATCGAATTCAATTAGAATGT
TAGGATCACAGTCACCGCTTATACAAGCATATGGACTAGTTATTTTACAACAGCCAGACA
TTAAGGTAAATGCGATGAGTAGTTTGACGAATCATCAAAAGTTTGCAAAGGCAAATGTAA
GAGAGTGGATTGATGAATATAATCCGAAGCTAATTGACTTAAATCAAGAGATGATGAGAT
ATAGTATTAGATTTAATAGTTATTACAGTAAGCTCTATGAACTAGCAGGAAACATAAATG
AAGATGAACAATCAAAAGCAGATTTTACAAATGCATATGGAAAACTACAATTGCAAGTAC
AAAGCATCCAAGAGAATATGGAGCAAGATTTATTAGAGTTAAATCGATTTAAAACGGTAT
TGGACAAAGATAGTAATAACTTATCAATTAAAGCTGATGAAGCAATAAAAACACTGCAAG
GATCAAGTGGAGATATTGTGAAATTAAGAGAAGATATTAAAAGAATTCAAGGGGAAATTC
AAGCTGAATTAACGACTATTTTGAATAGACCTCAAGAAATTATTAAAGGCTCTATTAATA
TCGGTAAACAAGTATTTACAATTACAAATCAAACTGCACAAACGAAAACAATTGATTTTG
TTTCTATCGGTACTTTAAGTAATGAAATTGTAAATGCTGCCGATAGTCAAACGAGGGAAG
CGGCTCTTCGTATTCAGCAAAAACAAAAAGAGCTCCTACCACTTATTCAAAAATTATCAC
AAACTGAAGCAGAGGCGACACAAATTACATTCGTTGAAGATCAAGTAAGTAGCTTTACAG
AACTAATTGACCGTCAAATTA
```

B.

ATGATAAAATAAACGGTTCTGGCAAGAAGCGTTGCACTAAGGCGACGCAACCATTAGTAA
AAGAACGAAACAAAATAGATCAACCTCCTTATGTATATGTATTACAATTATTCGAGGTTG
TTTCGTTCATAACCTCTTAAAAATAGAAAATTCTGATATTTTTAAAAGGATTGAAATATG
TTCAAGAAGAGTGGTAGCATCATCACCCTGACAGAAAAAGTTGCTTTTTCCCTAATATGA
AATATTGCATATAAAAATGAATTGATGCAAATGTAAAAGCTTATTATAATGAAAGCATAA
ATATACAACACATCTTAATACAGCAAAGAGGAGGAAGGAATATGGAAATCGCAATGGCAG
TTTTAAAATTTTTAGGTGGAGTAATTCCTTTAGTTCAAGAACTTTTAAAAGCGTTTATGT
AAGTTTATTCGTTAGCTATCATATATCCATCATTATTATATCTTACTAAAAAAAGAACGG
GGTGATTTTTTCTGAATATT**ATG**AAAAATTTTAAGGGAAGAAAGTTCCTTACATGTGTAT
TAGTTAGTCTATGCACTCTTAATTATTCATCTATTTCTTTCGCAGAAACACAAGCCGGTC
ATGCAAATGATATAACTAAAAATGCTAGTAGCATTGATACTGGTATAGGGAATCTTACAT
ATAATAATCAAGAGGTTTTAGCTGTAAATGGTGATAAAGTAGAGAGTTTTGTTCCGAAAG
AAAGTATCAATTCAAATGGTAAATTTGTAGTAGTGGAACGTGAGAAAAAATCACTTACAA
CGTCACCAGTCGATATTTCGATTATTGATTCTGTAGCGAATCGTACGTATCCAGGAGCTG
TACAACTTGCCAATAAAGCTTTTGCAGACAATCAACCGAGTTTATTAGTGGCTAAGAGAA
AGCCTTTGAATATTAGTATAGACTTACCTGGTATGAGAAAAGAAAATACAATCACTGTCC
AAAATCCGACATATGGTAATGTGGCTGGAGCAGTAGATGATTTAGTATCTACTTGGAATG
AAAAGTATTCCACAACACATACGTTACCTGCAAGAATGCAGTATACAGAATCTATGGTGT
ATAGTAAAGCACAAATCGCAAGTGCTCTTAATGTTAATGCTAAATATCTTGATAACAGTC
TAAATATTGACTTTAATGCAGTTGCAAATGGAGAAAAGAAAGTGATGGTGGCCGCATATA
AACAAATATTTTATACTGTAAGTGCTGAACTACCTAACAATCCATCTGATCTCTTTGATA
ATAGTGTTACTTTTGGTGAGTTAACTCGTAAAGGAGTAAGCAATTCGGCTCCACCTGTTA
TGGTTTCAAATGTAGCTTATGGTAGAACGGTTTATGTAAAATTAGAAACAACATCTAAGA
GTAAAGATGTACAAGCTGCTTTTAAAGCTTTACTTAAGAATAACAGCGTTGAAACGAGTG
GGCAGTACAAAGATATTTTTGAAGAAAGTACCTTTACTGCTGTCGTATTAGGTGGAGATG
CGAAAGAGCATAACAAGGTTGTTACAAAAGATTTCAATGAAATCCGAAATATCATTAAAG
ATAATGCTGAATTAAGTTTTAAAAATCCAGCATATCCAATTTCATATACAAGCACGTTCT
TAAAAGATAATGCAACTGCTGCTGTTCATAACAATACAGATTATATCGAGACGACAACTA
CAGAATATTCAAGTGCTAAAATGACACTTGATCATTACGGTGCTTATGTTGCTCAATTTG
ATGTATCTTGGGATGAATTCACATTTGATCAAAATGGGAAAGAAATACTAACACATAAAA
CATGGGAAGGTAGTGGCAAAGACAAAACAGCTCATTATTCTACAGTAATCCCTCTTCCAC
CAAATTCAAAAAATATAAAAATCGTAGCAAGAGAATGTACAGGTCTTGCATGGGAATGGT
GGAGAACAATTATTAATGAACAAAATGTTCCATTAACAAATGAAATAAAGGTTTCAATTG
GAGGAACAACATTATACCCAACGGCTAATATTAGTCAT**TAG**

C.

AACATTAACATCCGGTACGATTTTTTATAGTACAGTTGAAGGATTACGTCCTCTTGACGC
TTTATATTTTAGTGTGGTCACGTTGACGACTGTCGGTGATGCTAATTTTAGCCCGCAAAC
TGATTTTGGAAAGATATTTACAATCTTATACATATTTATTGGGATTGGATTAGTATTTGG
ATTTATTCATAAGTTGGCAGTTAATGTACAACTGCCGAGTATATTATCGAATAGAAAAAA
AGAGTAAAGCATGTGGATGCTTTACTCTTTTTTTACATTAGTTTAGATAAGCCTTAATAA
TAGTTTATTAGAATGAAAGTGTATTCATTCATTATATTCACTGTGTATAAAGTTATAATG
ATATGAAAATCTGCATATTTTAATTTAGTGATAGAAATTTCGTGAAAGGTGGGATATTCT
AGTCATAGGTTAACCGGACGACATCATAGGATCCTAACAAAAAGTTTACAATAATTCGAT
TATAAA**ATG**GAGGATTTCAGATGAAAAAGAAAGTACTTGCTTTAGCAGCAGCTATTACAG
TAGTAGCTCCTTTACAAAGCGTTGCATTTGCTCATGAAAACGATGGGGGAAGTAAAATAA
AAATAGTTCACCGCTGGTCTGCTGAAGATAAACATAAAGAAGGCGTAAATTCTCATTTAT
GGATTGTAAACCGTGCGATTGATATTATGTCTCGCAATACAACACTTGTAAAACAAGATC
GAGTTGCACAATTAAATGAATGGCGTACGGAGTTAGAGAACGGTATTTATGCTGCTGACT
ATGAAAATCCTTATTATGATAATAGCACATTTGCTTCACATTTCTATGATCCAGACAATG
GAAAAACATATATTCCATTTGCAAAGCAGGCAAAAGAAACTGGCGCTAAATATTTTAAAT
TAGCTGGTGAATCATATAAAAATAAAGATATGAAACAAGCATTCTTCTATTTAGGATTAT
CTCTTCATTATTTAGGAGATGTAAACCAACCGATGCATGCAGCAAACTTTACAAATCTTT
CGTATCCACAAGGATTCCATTCTAAATATGAAAACTTTGTAGATACGATAAAAGATAATT
ATAAAGTGACGGATGGAAATGGATATTGGAACTGGAAAGGTACAAATCCAGAAGAGTGGA
TTCATGGAGCGGCAGTAGTAGCGAAACAAGATTACTCTGGAATTGTAAATGATAATACGA
AAGATTGGTTCGTAAAAGCAGCTGTGTCACAAGAATATGCAGATAAATGGCGCGCTGAAG
TTACGCCAATGACAGGTAAGCGATTAATGGATGCACAACGTGTTACTGCTGGATACATTC
AGCTTTGGTTTGATACGTACGGAGATCGT**TAA**

Figure 1. Putative CRE sites. Nucleotides sequences of BCAH187_A1995 (**A**), BCAH187_A3327 (**B**), BCAH187_AO804 (**C**) and their 500-bp upstream region. CRE sites are underlined. ORF sequences encoding NheA, CLO and PlC toxins are written in blue.
